# Recent Advances in the Elucidation of Frataxin Biochemical Function Open Novel Perspectives for the Treatment of Friedreich’s Ataxia

**DOI:** 10.3389/fnins.2022.838335

**Published:** 2022-03-02

**Authors:** Beata Monfort, Kristian Want, Sylvain Gervason, Benoit D’Autréaux

**Affiliations:** Université Paris-Saclay, CEA, CNRS, Institute for Integrative Biology of the Cell (I2BC), Gif-sur-Yvette, France

**Keywords:** frataxin, iron-sulfur cluster, persulfide, Friedreich’s ataxia, therapy

## Abstract

Friedreich’s ataxia (FRDA) is the most prevalent autosomic recessive ataxia and is associated with a severe cardiac hypertrophy and less frequently diabetes. It is caused by mutations in the gene encoding frataxin (FXN), a small mitochondrial protein. The primary consequence is a defective expression of FXN, with basal protein levels decreased by 70–98%, which foremost affects the cerebellum, dorsal root ganglia, heart and liver. FXN is a mitochondrial protein involved in iron metabolism but its exact function has remained elusive and highly debated since its discovery. At the cellular level, FRDA is characterized by a general deficit in the biosynthesis of iron-sulfur (Fe-S) clusters and heme, iron accumulation and deposition in mitochondria, and sensitivity to oxidative stress. Based on these phenotypes and the proposed ability of FXN to bind iron, a role as an iron storage protein providing iron for Fe-S cluster and heme biosynthesis was initially proposed. However, this model was challenged by several other studies and it is now widely accepted that FXN functions primarily in Fe-S cluster biosynthesis, with iron accumulation, heme deficiency and oxidative stress sensitivity appearing later on as secondary defects. Nonetheless, the biochemical function of FXN in Fe-S cluster biosynthesis is still debated. Several roles have been proposed for FXN: iron chaperone, gate-keeper of detrimental Fe-S cluster biosynthesis, sulfide production stimulator and sulfur transfer accelerator. A picture is now emerging which points toward a unique function of FXN as an accelerator of a key step of sulfur transfer between two components of the Fe-S cluster biosynthetic complex. These findings should foster the development of new strategies for the treatment of FRDA. We will review here the latest discoveries on the biochemical function of frataxin and the implication for a potential therapeutic treatment of FRDA.

## Introduction

The severe neurological disease Friedreich’s Ataxia (FRDA) is the most frequent hereditary autosomal recessive ataxia with an incidence of about 30,000 in the Caucasian population (people with European ancestry) and a carrier rate near 1:100 (Delatycki et al., 2000; Synofzik and Németh, 2018). The disease was first described by Dr. Nikolaus Friedreich in the mid-19th century, who documented the pathology of several patients suffering from “degenerative atrophy of spinal posterior column” and heart problems related to hypertrophic cardiomyopathy, which was confirmed by later studies to be the dominant cause of death characterized by left ventricular hypertrophy, smaller left ventricular diastolic diameters and volumes, increased wall thicknesses and arrhythmia (Friedreich, 1863; Tsou et al., 2011). Patients display signs of cerebellar, limb and sensory ataxia with symptoms onset generally during the first two decades of life (Cook and Giunti, 2017; Smith and Kosman, 2020). Diabetes, blindness and deafness are additional phenotypes that appear in the course of the disease but with a different incidence frequency, onset and progression (Parkinson et al., 2013; Lynch et al., 2021).

Friedreich’s ataxia is caused by mutations on chromosome 9, in the gene coding for the frataxin protein (FXN) (Chamberlain et al., 1988; Campuzano et al., 1996). The highest amount of frataxin mRNA is present in the heart, spinal cord, liver, skeletal muscle, and pancreas, which are also the most affected sites in patients with FRDA (Campuzano et al., 1996; Jiralerspong et al., 1997). The vast majority of patients (∼96%) have abnormal GAA-triplet expansions of different length on both alleles of the first intron of the frataxin gene, leading to a drastic decline in the transcription of frataxin mRNA and consequently reduced levels of frataxin (Yandim et al., 2013; Gottesfeld, 2019). About 4% of patients are heterozygotes for a GAA-triplet expansion on one allele and a point mutation or an insertion/deletion in the FXN gene on the other one (Santos et al., 2010; Delatycki and Corben, 2012; Galea et al., 2016). Frataxin levels are also decreased in compound heterozygous patients together with expression of dysfunctional forms of frataxin. The amount of remaining frataxin in FRDA patients ranges from 30 to 2%, depending on the extent of the silencing that is linked to the number of GAA-triplet repeats (Campuzano et al., 1997; Lazaropoulos et al., 2015). The onset and the severity of the neurological features also correlate with the number of GAA-triplet repeats; this is less clear for the non-neurological symptoms (diabetes, cardiomyopathy, scoliosis, and pes cavus) and atypical features (Campuzano et al., 1996; Dürr et al., 1996; Filla et al., 1996; Montermini et al., 1997; Lazaropoulos et al., 2015; Reetz et al., 2016, 2021; Indelicato et al., 2020). Intron 1 of the FXN gene typically contains up to about 30 GAA expansions. The pathological late onset starts at about 40–60 GAA on the shorter allele with a mean value of about 300 GAA. As the number of GAA increases, the onset of the disease occurs earlier, with an average value of approximately 600 GAA and up to 1,700 characterizing early onset. This suggests a direct link between the level of remaining frataxin and the severity of the neurological features (onset and state of health).

Major breakthroughs in exploring frataxin’s function came from studies of the cellular phenotypes of FRDA patient tissues (Rotig et al., 1997) and animal and cellular FRDA models from human (Liu et al., 2011), mouse (Cossee et al., 2000; Puccio et al., 2001; Miranda et al., 2002), drosophila (Calap-Quintana et al., 2018; Russi et al., 2020) and yeast (Babcock et al., 1997; Foury and Cazzalini, 1997; Duby et al., 2002; Muhlenhoff et al., 2002). The first yeast model containing a knockout mutation of Yfh1, the yeast frataxin homolog, resulted in mitochondrial iron accumulation, which led to the conclusion that Yfh1 is involved in mitochondrial iron homeostasis (Babcock et al., 1997; Foury and Cazzalini, 1997). Further observations uncovered that abnormal frataxin production led to a marked decrease in enzymatic activities of mitochondrial and extra-mitochondrial iron-sulfur (Fe-S) cluster enzymes (Foury and Cazzalini, 1997; Rotig et al., 1997; Wilson and Roof, 1997; Puccio et al., 2001; Duby et al., 2002; Muhlenhoff et al., 2002; Lu and Cortopassi, 2007; Martelli et al., 2007). Fe-S clusters are protein cofactors providing catalytic activities to numerous enzymes with essential functions in ATP production (Stiban et al., 2016), Krebs cycle (Switzer, 1989), redox catalysis (Bauerle et al., 2015; Kimura and Suzuki, 2015), protein (Kispal et al., 2005) and DNA (Torrents et al., 2002; Zhang et al., 2011, 2014) synthesis, signaling (Crack et al., 2014) as well as DNA maintenance (Fuss et al., 2015; Shi et al., 2021). Any defect in their biosynthesis thus lead to metabolic defects affecting energy production and many other cellular functions (Beilschmidt and Puccio, 2014; Stehling et al., 2014; Alsina et al., 2018b; Cardenas-Rodriguez et al., 2018). In addition to the Fe-S cluster biosynthesis defect, sensitivity to oxidative stress and decline in enzymatic activities of heme enzymes were also observed (Foury and Cazzalini, 1997; Wilson and Roof, 1997; Lesuisse et al., 2003; Lange et al., 2004; Schoenfeld et al., 2005; Al-Mahdawi et al., 2006; Lu and Cortopassi, 2007). Oxidative stress in FRDA patients is thought to result from the production of reactive oxygen species catalyzed by free iron accumulating in mitochondria (Fenton reaction) and from impaired oxidative stress signaling by the master regulator NRF2 (NF-E2-related factor) allowing reactive oxygen species to accumulate (Alsina et al., 2018a). This array of intricate phenotypes made it difficult to assign the primary function of frataxin, until it became apparent that frataxin is directly involved in the biosynthesis of Fe-S clusters and that the other phenotypes appear consequently to the Fe-S cluster biosynthesis defect (Pastore and Puccio, 2013).

Several therapeutic strategies targeting either the primary or secondary defects of FRDA are under development following two main directions: (1) restoration of FXN levels using gene expression modulators, protein stabilizers and gene therapy and (2) alleviation of secondary cellular defects such as mitochondrial functions, iron accumulation, oxidative stress and ferroptosis (Clay et al., 2019; Zesiewicz et al., 2020; Lynch and Farmer, 2021; Ocana-Santero et al., 2021; Pallardó et al., 2021; Yang et al., 2021). Hereby, pharmacologic approaches including iron-chelators, antioxidants, NRF2 activators, ferroptosis inhibitors and molecules improving mitochondrial functions have reached clinical trials. Promising results were also reported with frataxin expression activators and gene therapy. A third strategy not yet explored would be to replace FXN using FXN-mimics to target the primary consequence of FRDA. However, this strategy is still unexploited as only recently the enzymatic function of frataxin in Fe-S cluster biosynthesis has become clear. This review summarizes and critically analyses the different proposed functions of frataxin in iron metabolism, heme and Fe-S cluster biosynthesis: iron storage, iron chaperone, gate-keeper of detrimental Fe-S cluster biosynthesis over activity, sulfide production stimulator or sulfur transfer accelerator (Srour et al., 2020). The latter is the most established function and could serve as a new direction to test potential therapeutics restoring Fe-S cluster biosynthesis in patients with FRDA.

## The Cellular Phenotypes of Friedreich’s Ataxia Point to a Key Role of Frataxin in Iron Metabolism

When the mutations responsible for FRDA were identified in the locus encoding the frataxin protein (FXN), its molecular function was not known (Campuzano et al., 1996). Soon after, orthologs of human FXN were identified in other organisms, such as Yfh1 in yeast and CyaY in bacteria, which has greatly contributed to the elucidation of FXN’s function. The first clue on the molecular function of FXN came from the analysis of the cellular phenotypes in FRDA patient tissues along with animal and cellular models. Among the variety of phenotypes, the most prominent and recurrent ones are the Fe-S cluster and heme deficits, iron accumulation within mitochondria, loss of mitochondrial DNA and a higher sensitivity to oxidative stress (Sanchez-Casis et al., 1976; Babcock et al., 1997; Foury and Cazzalini, 1997; Rotig et al., 1997; Wilson and Roof, 1997; Puccio et al., 2001; Muhlenhoff et al., 2002; Lesuisse et al., 2003; Schoenfeld et al., 2005). However, the severity of these phenotypes differs substantially from one organism to another and depends on the cell-type. For instance, iron accumulation, oxidative stress and heme deficiency are not systematically observed and are tissue-specific (Cossee et al., 2000; Becker et al., 2002; Muhlenhoff et al., 2002; Seznec et al., 2005; Steinkellner et al., 2017). Thereby, no definitive conclusions were drawn on the exact function of frataxin based on these observations. Moreover, it was unclear at the time which cellular phenotypes correspond to either primary or secondary effects due to the lack of FXN.

Nonetheless, these phenotypes were pointing to a key function of FXN in iron metabolism. Fe-S clusters and heme are iron-containing protein cofactors providing enzymatic activities to a multitude of proteins involved in essential biological functions. Among the multifaceted functions of Fe-S proteins are electron transfer for ATP production in mitochondria (Stiban et al., 2016) and DNA synthesis (Zhang et al., 2014), redox catalysis in numerous metabolic pathways by the radical SAM superfamily (Bauerle et al., 2015; Kimura and Suzuki, 2015), non-redox catalysis in the Krebs cycle (Switzer, 1989; Stiban et al., 2016) and protein synthesis by ribosomes (Kispal et al., 2005), iron donation (Zhang et al., 2011), sulfur donation (Lotierzo et al., 2005; Fugate and Jarrett, 2012; Mulliez et al., 2017), signaling (Crack et al., 2014) and maintenance of genome integrity (Fuss et al., 2015; Shi et al., 2021). Heme containing enzymes are also involved in electron transfer for ATP production (Santucci et al., 2019), signaling (Shimizu et al., 2019) and redox catalysis (Flohé, 2020), but also oxygen transport (Sen Gupta, 2019), xenobiotic detoxification (Esteves et al., 2021) and oxidative stress defense (Fugate and Jarrett, 2012; Aratani, 2018). It is interesting to note that in yeast strains deleted for the *fxn* gene, FXN is totally absent but Fe-S clusters and hemes are still produced (Babcock et al., 1997; Duby et al., 2002; Muhlenhoff et al., 2002; Lange et al., 2004). This suggests that FXN is a regulator rather than an obligatory enzyme.

## Iron Storage Function

The presence of granular iron deposits in the mitochondria of cardiomyocytes was one of the first findings of iron metabolism dysregulation in FRDA patients (Sanchez-Casis et al., 1976; Lamarche et al., 1980; Martelli and Puccio, 2014), which very quickly led to the suspicion that frataxin could function as an iron storage protein (Adamec et al., 2000; Cossee et al., 2000; Gakh et al., 2002). Although iron deposits were not observed in the cerebellum, hallmarks of iron dysregulation such as the modification of the expression of the iron-regulated proteins transferrin receptor 1 (TFR1), ferritins (FRTs) and ferroportin (FPN) are manifest in neurons (Martelli and Puccio, 2014). A first hypothesis on the molecular function of frataxin emerged when purified Yfh1, the yeast frataxin, was found to directly bind iron and to oligomerize in a ferritin-like shape hosting thousands of iron atoms (Adamec et al., 2000). An iron storage function was thus proposed for frataxin.

### Overall Structure of Monomeric Frataxins

The full-length human frataxin (FXN^210^, 23 kDa) is translated as a precursor protein in the cytosol and targeted to mitochondria using a targeting sequence in its N-terminal domain that is cleaved upon import into mitochondria by the mitochondrial processing peptidase (MPP) in an unusual two-step process (Koutnikova et al., 1998; Condò et al., 2007; Schmucker et al., 2008). During the first step, the precursor is cleaved into an intermediate form (FXN^42–210^, 18 kDa) that is subsequently shortened to the mature form (FXN^81–210^, 14 kDa) in the second step. A minor fraction of frataxin also exists as isoforms with extra-mitochondrial localization in the cytoplasm and the nucleus that originate from alternative codon initiation and splicing events (Pianese et al., 2002; Condo et al., 2006; Xia et al., 2012; Abruzzo et al., 2013; Pérez-Luz et al., 2015; Guo et al., 2018; Agrò and Díaz-Nido, 2020; Weng et al., 2020). Two isoforms have been characterized in human that both contain an elongated N-terminus, FXN II (FXN^76–210^, 15 kDa) and FXN III (FXN^1–5,53–210^, 18 kDa), with an N-acetyl modification in the case of FXN II (Xia et al., 2012; Guo et al., 2018). They have distinct tissue distribution with FXN II mRNA more abundant in the cerebellum and FXN III mRNA in the heart (Xia et al., 2012) and N-acetylated FXN^76–210^ the predominant form in erythrocytes (Guo et al., 2018). Extra-mitochondrial frataxin isoforms apparently contribute to cellular fitness as was shown in cell survival (Condo et al., 2006) and respiration assays (Agrò and Díaz-Nido, 2020), but their exact biochemical functions are still unclear. In mouse, a truncated cytosolic isoform (FXN^79–207^) is the predominant form, suggesting that it might contribute to the FRDA phenotypes in this model (Weng et al., 2020). However, this isoform is not present in human subjects. In human, the proportions of FXN II and III vs. the canonical mature mitochondrial form (FXN^81–210^) have not been determined, except in the heart of FRDA patients, where the canonical FXN is the predominant form (Weng et al., 2020), which questions the role of the isoforms.

**FIGURE 1:**
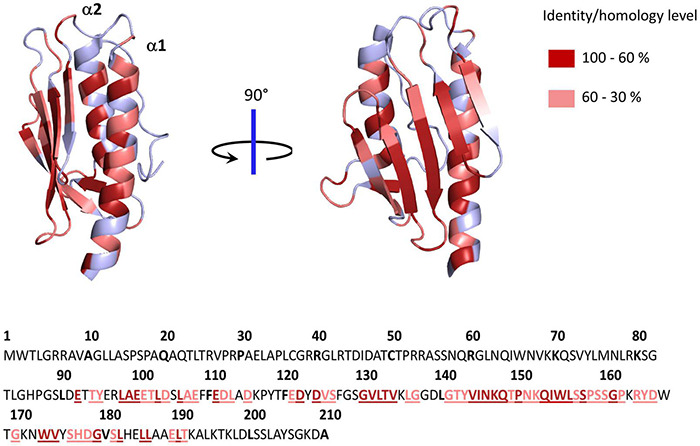
Structure of human frataxin (FXN) mapping conserved amino acids. Structure of human FXN (PDB code 1EKG) highlighting conserved amino acids from alignment of 50 eukaryotic and 50 prokaryotic frataxins (Supplementary Figure 1). Below is the sequence of full length human frataxin. Amino acids colored in dark red have identity/homology percentage between 100 and 60% and those in light red between 60 and 30%.

The structures of several frataxin proteins from various organisms have been solved, showing a remarkably conserved structure (Cho et al., 2000; Dhe-Paganon et al., 2000; Musco et al., 2000; Adinolfi et al., 2002; He et al., 2004). The mature form folds into an α-β sandwich structural motif composed of a six to seven stranded β-sheet forming a flat surface on one side and two α-helix on the other side (Figure 1). A high number of conserved acidic amino acids (Supplementary Figure 1) are present in the first α-helix (α1) and the edge of the following β-sheet, which defines an acidic ridge with a negatively charged surface (Figure 1). A patch of highly conserved amino acids are also gathered in the middle of the β-sheet including a strictly conserved tryptophan at position 155 (Figure 1). Eukaryotic frataxins also contain a longer N-terminal domain of about 10 amino acids (81–92) that is unstructured and poorly conserved (Adinolfi et al., 2002; Prischi et al., 2009).

### Iron-Dependent Oligomerization

Recombinant frataxin from *S. cerevisiae*, *E. coli* and human have been shown to self-assemble into higher-order oligomers that bind iron (Adamec et al., 2000; Adinolfi et al., 2002; Gakh et al., 2002; O’Neill et al., 2005). Titration of Yfh1 with increasing iron concentrations under aerobic conditions leads to the stepwise assembly of trimers into multimers of 48–60 subunits sequestering over 2,000–3,000 atoms of iron within iron cores of 2–4 nm (Adamec et al., 2000; Gakh et al., 2002). The assembly of Yfh1 was further shown to be driven by iron oxidation and mineralization of Fe^3+^ ions. Conversely, iron core reduction resulted in protein disassembly. A similar iron-dependent oligomerization process was later on reported for the bacterial frataxin CyaY (Adinolfi et al., 2002). Human FXN was also found to bind multiple iron ions and to oligomerize, but in an iron independent manner *via* stable interactions mediated by its extended N-terminal region (Cavadini et al., 2002). Based on these data, frataxin was proposed to function as an iron storage protein maintaining iron bio-availability for Fe-S cluster and heme biosynthesis. Nevertheless, the physiological relevance of this iron-dependent oligomerization process has been questioned. First, only immature precursors of human FXN (FXN^42–210^, FXN^55–210^) were shown to oligomerize; furthermore, in iron-independent manners (Musco et al., 2000; Adinolfi et al., 2002; Cavadini et al., 2002; Schmucker et al., 2008; Gakh et al., 2010). Iron-dependent assembly of mature human FXN was only observed under strongly destabilizing conditions at 57°C (Adinolfi et al., 2002). This behavior might be linked to the higher thermal stability of human FXN compared to yeast and bacterial ones (Adinolfi et al., 2004), hence suggesting that oligomerization may be related to protein denaturation. However, a recent study estimated the internal temperature of mitochondria close to 50°C, which could promote oligomerization of mature frataxin (Chrétien et al., 2018). Moreover, recent studies revived the potential of human frataxin to oligomerize in an iron-dependent manner. This was observed by dynamic light scattering (DLS) but oligomerization occurred solely at a large iron excess (iron:FXN molar ratio of 10:1) with significantly slow kinetics (60 min for complete oligomerization) (Ahlgren et al., 2017). Unfortunately, a high-resolution structure of the human mature FXN^81–210^ oligomer is still lacking. Many studies reported iron-binding properties of monomeric frataxin proteins (Campbell et al., 2021). A total of twelve aspartate and glutamate residues along the conserved acidic ridge were found to bind iron but no structurally defined iron-binding site could be identified. A crystallographic structure of a Yfh1 trimer containing iron was generated by soaking the crystals of the apo-FXN trimer into an iron solution (Soderberg et al., 2013). However, neither of the residues of the acidic ridge were binding iron. Instead, the iron ion was found in an unusual configuration, apparently held by only two oxygen atoms from the amide moieties of Thr118 and Ala133 at distances of about 4 Å, a binding mode inconsistent with metal coordination. This strengthens the idea that iron binding to frataxin is non-specific and mediated by electrostatic interactions.

Further evidence against a role of frataxin as an iron storage protein under physiological conditions came from *in vivo* studies. In yeast cells, a Yhf1 mutant unable to perform self-assembly fully complemented a Δ*yfh1* strain, clearly indicating that frataxin oligomerization is not needed for its cellular function (Aloria et al., 2004). In a yeast strain accumulating iron in mitochondria, Yfh1 overexpression was neither able to prevent iron accumulation nor modify the nature of the iron minerals deposit (Seguin et al., 2010). Finally, in different mouse models of FRDA, a chronological analysis of phenotypic appearance showed that the Fe–S cluster deficit appears very early in the course of the disease, followed by the cardiac symptoms, oxidative stress and only afterward iron deposition, thus pointing to iron accumulation as a late, secondary effect of the Fe-S cluster defect (Puccio et al., 2001; Martelli and Puccio, 2014; Poburski et al., 2016).

In conclusion, these data have refuted the iron storage function of FXN. The accumulation of iron is in fact not a specific feature of frataxin deficiency but of defects in the early stages of the Fe-S cluster biosynthetic pathway due to iron misuse (Lill et al., 2012; Muhlenhoff et al., 2015). Thereby, it is now generally accepted that iron accumulation is a consequence of the Fe-S cluster defect, thus indicating that FXN is not a general ferritin-like iron storage protein but operates more directly in Fe-S cluster and/or heme biosynthesis.

## The Primary Role of Frataxin Is in Iron-Sulfur Cluster Biosynthesis, Not in Heme

While the iron storage function was disproved, frataxin was proposed to operate as an iron donor/chaperone for heme and Fe-S cluster biosynthesis. Direct interactions between FXN and ferrochelatase, the enzyme inserting iron into protoporphyrin IX (PPIX) at the last step of heme synthesis, were detected *in vitro* (Lesuisse et al., 2003; He et al., 2004; Yoon and Cowan, 2004; Soderberg et al., 2016). However, the interaction with ferrochelatase was not confirmed *in vivo*, in neither mammalian (Schmucker et al., 2011) nor yeast cells (Lange et al., 2004). Moreover, the insertion of iron into PPIX was not impaired in frataxin depleted cells from yeast (Muhlenhoff et al., 2002) and mammal (Schoenfeld et al., 2005). Rather, the defect in heme synthesis was explained by an inhibition of ferrochelatase activity by a still ill-defined mechanism as well as an increased level of zinc leading to the accumulation of Zn-PPIX (Lange et al., 2004; Schoenfeld et al., 2005). In erythroid progenitors from FRDA patients, heme biosynthesis was not altered (Morgan et al., 1979; Steinkellner et al., 2017). Altogether, these data suggest that FXN is not involved in heme biosynthesis. A defect in the insertion of iron into PPIX was reported in yeast, but in a strain where the *yfh1* gene was deleted, thus totally lacking Yfh1, which may correspond to more stringent conditions allowing the accumulation of secondary defects (Lesuisse et al., 2003). This suggests that the ferrochelatase defect is a late indirect effect of frataxin deficiency. This was confirmed in a human embryonic kidney cell line through chronological investigations of the phenotypic defects caused by progressive FXN depletion revealing that the heme defect occurs much later than the Fe-S cluster defect and oxidative stress (Lu and Cortopassi, 2007). Moreover, with the notable exception of erythroid cells, the defects in heme biosynthesis do not lead to conspicuous iron accumulation in mitochondria, in contrast to both frataxin and Fe-S cluster deficiencies (Crisp et al., 2003; Chung et al., 2012; Lill et al., 2012; Muhlenhoff et al., 2015). Finally, a causal relationship between the Fe-S cluster defect and heme deficiency might exist in human and the other organisms where the ferrochelatase holds a [2Fe2S] cluster (Weerth et al., 2021). Although the function of this Fe-S cluster is still elusive, heme biosynthesis might be directly affected by the Fe-S cluster defect in these organisms.

In contrast, several independent studies reported reliable interactions both *in vitro* and *in vivo* between frataxin and two components of the Fe-S cluster assembly machinery, the scaffold protein ISCU and the cysteine desulfurase NFS1 (Gerber et al., 2003; Yoon and Cowan, 2003; Ramazzotti et al., 2004; Foury et al., 2007; Wang and Craig, 2008; Schmucker et al., 2011). Moreover, several *in vitro* reconstitutions of Fe-S cluster assembly machineries from a diverse range of organisms have further unveiled a functional role of frataxins in Fe-S cluster biosynthesis (Srour et al., 2020). It is now commonly accepted that the primary function of frataxin is the regulation of Fe-S cluster biogenesis whereas the heme defect, iron accumulation and oxidative stress are consequences of the Fe-S cluster defect. However, the exact function of FXN in Fe-S cluster biosynthesis remained controversial until very recently.

## Specialized Role of Frataxin in Iron-Sulfur Cluster Biosynthesis

### Overview of the Iron-Sulfur Cluster Assembly Process

**FIGURE 2:**
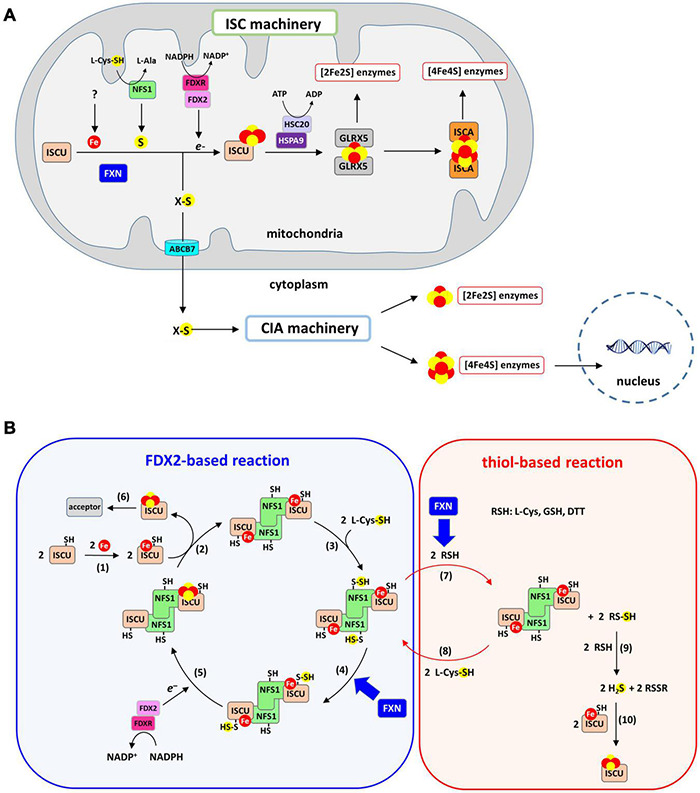
Iron-sulfur (Fe-S) cluster biogenesis pathway. **(A)** Fe-S cluster biosynthesis is initiated in mitochondria by the ISC machinery that encompasses the scaffold protein ISCU on which [2Fe2S] clusters are assembled, the cysteine-desulfurase NFS1 that provides sulfur in the form of a cysteine-bound persulfide and FDX2/FDXR that reduces the persulfide to sulfide. Frataxin accelerates the formation of the [2Fe2S] cluster on ISCU. The [2Fe2S] cluster is transferred onto GLRX5 with assistance from the ATP-dependent chaperones HSC20 and HSPA9, and then to recipient apo-proteins. Conversion to a [4Fe4S] cluster from the [2Fe2S] cluster is achieved by the ISCA proteins. The ISC machinery generates a sulfur-containing compound (compound X) that is exported by the ABCB7 transporter to the CIA machinery. [4Fe4S] clusters are assembled by the CIA machinery and delivered to their cytoplasmic and nuclear protein acceptors. **(B)** Mechanism of Fe-S cluster biosynthesis by the ISC machinery: (1) a ferrous iron is inserted into the assembly site of ISCU, (2) two Fe-ISCU form a heterodimer with a dimer of NFS1 (ACP and ISD11 that bind NFS1 are omitted for clarity), (3) NFS1 catalyzes the formation of persulfide on its catalytic cysteine, (4) the persulfide of NFS1 is transferred to ISCU and FXN accelerates this reaction, (5) FDX2/FDXR reduces the persulfide into sulfide, leading to formation of a [2Fe2S] cluster most likely by dimerization of ISCU, (6) the [2Fe2S] cluster carried by ISCU is transferred to acceptor proteins. Step 1–6 describe the FDX2-based reaction. A non-physiological process called the thiol-based reaction allows formation of Fe-S cluster *in vitro* in the absence of FDX2/FDXR *via* the following steps: (7) thiols (RSH) such as DTT, L-cysteine or GSH reduces the persulfide of NFS1, which leads to formation of persulfidated thiols (RSSH). FXN accelerates this persulfide cleavage by thiols. (8) the persulfide of NFS1 is regenerated by reaction with L-cysteine, (9) RSSH reacts with a second thiol to form free sulfide in the form of H_2_S alongside oxidized RSSR, (10) free sulfide slowly incorporates into Fe-ISCU to form [2Fe2S] and [4Fe4S] clusters by an unknown mechanism, with DTT favoring formation [4Fe4S] clusters.

Strikingly, although eukaryotic frataxins are mitochondrial proteins, they are involved in both mitochondrial and extra-mitochondrial Fe-S cluster biogenesis, thus pointing to a pivotal role in this process (Figure 2A; Muhlenhoff et al., 2002; Lu and Cortopassi, 2007; Martelli et al., 2007). Indeed, in eukaryotes, the mitochondrion plays a central role in cellular Fe-S cluster biosynthesis (Lill and Freibert, 2020; Srour et al., 2020). This process is initiated in mitochondria by the Fe-S cluster assembly machinery (ISC) on a scaffold protein called ISCU. The ISC machinery synthesizes [2Fe2S] clusters on ISCU that are transferred to recipient apo-proteins (Gervason et al., 2019). A set of specialized proteins also use [2Fe2S] clusters as building blocks for the assembly of the cubane [4Fe4S] cluster on ISCA scaffold proteins (Muhlenhoff et al., 2011; Beilschmidt et al., 2017; Weiler et al., 2020). Additionally, the ISC machinery synthesizes a sulfur-containing compound of yet-undefined nature, named compound X, that is exported to the cytoplasm to sustain the production of cytoplasmic and nuclear Fe-S clusters by the cytosolic iron-sulfur cluster assembly machinery (CIA). Compound X has been proposed to be either a poly sulfur molecule or a [2Fe2S] cluster ligated by small molecules, possibly glutathione (GSH) (Schaedler et al., 2014; Li and Cowan, 2015; Pandey et al., 2019; Lill and Freibert, 2020). Frataxin is specifically involved in the synthesis of the [2Fe2S] clusters by the ISC core machinery and most likely compound X synthesis as well, since the lack of frataxin leads to a Fe-S cluster defect in the cytoplasm (Lu and Cortopassi, 2007; Martelli et al., 2007).

A recent *in vitro* reconstitution of the complete ISC machinery performed in our laboratory using recombinant mouse proteins has shed new light on the mechanism of Fe-S cluster biosynthesis and the functional role of FXN (Figure 2B, blue chart) (Parent et al., 2015; Gervason et al., 2019; Srour et al., 2020). Using a biochemical assay to track sulfur processing during the assembly process within the ISC machinery, we unraveled the Fe-S cluster assembly process step-by-step (Parent et al., 2015; Gervason et al., 2019, 2021). The synthesis of Fe-S clusters is initiated by the insertion of a Fe^2+^ ion into the assembly site of the ISCU scaffold protein (Figure 2B, reaction 1). Sulfur is then provided by NFS1, a pyridoxal phosphate (PLP) dependent enzyme, in the form of a persulfide on its catalytic cysteine carried by a mobile loop. Two additional small proteins bind to NFS1, the LYR motif containing protein ISD11 and the acyl carrier protein ACP, which together regulate Fe-S cluster biosynthesis in response to acetyl-coA availability by stabilizing NFS1 (Van Vranken et al., 2018). NFS1 and ISCU form a heterodimeric complex (Figure 2B, reaction 2) in which the persulfide generated by NFS1 (Figure 2B, reaction 3) is transferred to ISCU when iron is present in the assembly site of ISCU (Figure 2B, reaction 4). Then, the ferredoxin FDX2, in complex with the NADPH dependent ferredoxin reductase FDXR, cleaves the persulfide into sulfide (S^2–^) and a [2Fe2S] cluster is formed, most likely by dimerization of ISCU (Figure 2B, reaction 5). At the final stage, the [2Fe2S] cluster is transferred to recipient apo-proteins by the ATP dependent chaperone/co-chaperone system HSPA9/HSC20 (Figure 2B, reaction 6). Several studies using reconstituted ISC machineries of mouse, human, yeast, and bacteria have reported a key function of frataxin in this process, but most of these reconstructions used incomplete ISC machineries, lacking FDX2 and FDXR (Figure 2B, red chart), which has led to a misinterpretation of the functional role of frataxin. We will first provide an overview of the results from these studies before introducing the data collected with the physiologically relevant reconstructions from complete ISC machineries.

### Iron Chaperone Function

Reconstitutions of the human and bacterial ISC machineries suggested that iron-loaded frataxin promotes the formation of Fe-S clusters by donating iron to ISCU (Yoon and Cowan, 2003; Layer et al., 2006; Colin et al., 2013). This iron chaperone function was attributed to the monomeric form of frataxin (Cook et al., 2006). However, the iron-binding site in ISCU was not characterized in these studies to ascertain that frataxin was actually donating iron to ISCU. The recent characterization of the iron binding site of mouse ISCU by spectroscopic techniques (electronic absorption, site directed mutagenesis, NMR, and Mössbauer) has enabled direct monitoring of iron insertion into ISCU (Gervason et al., 2019). It was found that ISCU directly binds iron in its assembly site without requirement of FXN or any other accessory proteins (Figure 2B, reaction 1). Another important feature of these studies that has previously been overlooked is the presence of a zinc ion in the assembly site of recombinant ISCU expressed in bacteria (Gervason et al., 2019). It is still unclear whether the zinc ion is present under physiological conditions or if it is an artifact of purification, but due to its high affinity for ISCU, it precludes iron binding. The zinc must be removed to allow iron binding. The ability of FXN to insert iron ions into zinc-containing ISCU was thus tested, but it was not able to exchange zinc with iron (Gervason et al., 2019).

**FIGURE 3:**
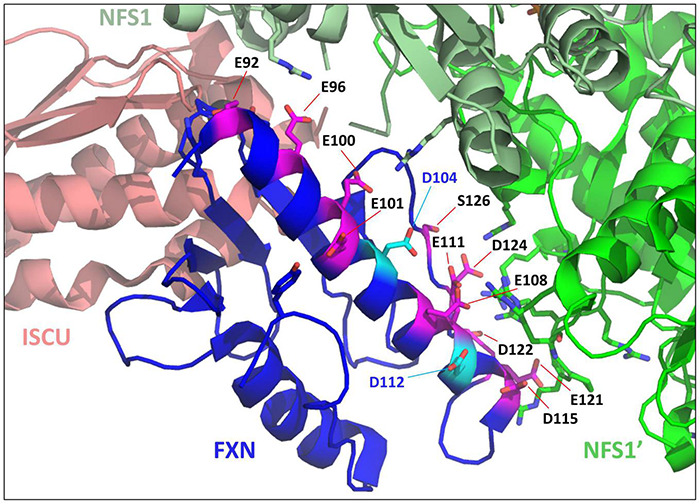
Mapping iron-binders amino acids of frataxin (FXN) within the NFS1-ISD11-ACP-Zn-ISCU-FXN complex. Structure of the human NFS1-ISD11-ACP-Zn-ISCU-FXN complex (PDB code 6NZU) highlighting the amino acids of FXN identified as iron-binders. The amino acids interacting with NFS1 (E96, E100, E108, E111, D115, E121, and D124) or important for the internal structure of FXN (E92, E101) are colored in pink and those not interacting with other amino acids (D104 and D112) are in cyan. ISCU is colored in salmon, the two subunits of the NFS1 dimer are in light green (NFS1) and dark green (NFS1’), FXN is in blue.

Moreover, as previously mentioned, the iron binding properties of frataxin point to a non-specific fixation mode (Pastore and Puccio, 2013). First, the number of iron bound to frataxin varied between studies (from one to seven), with Fe^2+^ and Fe^3+^ binding with similar affinities. The proposed iron-binding site involves several glutamate and aspartate residues from the conserved acidic ridge (Campbell et al., 2021). However, the structure of the complex between FXN and the NFS1-ISD11-ACP-ISCU complex (Fox et al., 2019) revealed that most of these amino acids are either directly involved in the interaction with NFS1 or in the internal structure of the protein (Figure 3). Only two of them, D104 and D112, are not interacting with other amino acids, but are also not strictly conserved across species and spatially too distant to form a structurally defined metal-binding site. Altogether, these data point to non-specific iron binding *via* electrostatic interactions, which is not consistent with the proposed function of frataxin as an iron chaperone.

In conclusion, these data invalidate the hypothesis that frataxin is an iron donor or iron chaperone for ISCU. The main hypothesis for the iron insertion process in ISCU is that it originates from labile iron pools, which are present in the cytoplasm as well as mitochondria where they maintain iron in its reduced form (Fe^2+^) bound to small molecules such as GSH (Hider and Kong, 2013; Lindahl and Moore, 2016; Philpott and Jadhav, 2019; Wofford et al., 2019).

### Stimulator/Inhibitor of Sulfide Production

The first evidence that FXN might be involved in sulfur donation rather than iron insertion came from studies of the bacterial ISC machinery (Adinolfi et al., 2009). *In vitro* reconstitutions of the bacterial ISC machinery were performed with recombinant IscU and IscS, the ortholog of NFS1, with DTT as a reductant instead of Fdx, and the homolog of FDX2 to cleave the persulfide into sulfide (Adinolfi et al., 2009). Hereafter, this type of reaction will be referred to as a thiol-based reaction (Figure 2B, red chart). Surprisingly, the enzymatic assays revealed that CyaY slows sulfide production by IscS and consequently Fe-S cluster production. This inhibitory effect was enhanced in the presence of free iron thus suggesting that the regulation by CyaY is iron-dependent. A function as a “gate-keeper” preventing uncontrolled production of sulfide ions in the presence of iron was then proposed for CyaY. However, the thiol-based assays performed with the human and mouse ISC systems revealed a completely reversed effect, with FXN stimulating the rate of sulfide production by NFS1, which was correlated with an acceleration of Fe-S cluster production (Tsai and Barondeau, 2010; Colin et al., 2013). The stimulatory effect of FXN was further shown to promote formation of [4Fe4S] clusters at the expense of [2Fe2S] clusters and FXN was proposed to facilitate iron entry concomitantly to its stimulatory effect on sulfide production (Colin et al., 2013). These data thus led to a controversy on the functional role of frataxins, with a protective effect of the bacterial frataxin on one hand, and a stimulatory function for the mammalian frataxin on the other hand.

Our laboratory later showed that the mechanism of stimulation by mammalian FXN relies on the acceleration of persulfide cleavage by DTT (Figure 2B, reaction 7), which increases sulfide release and Fe-S assembly by the thiol-based pathway (Parent et al., 2015). However, the thiol-based reaction cannot be considered as physiologically relevant and thus the significance of the stimulatory effect of FXN in this process is unclear. DTT and thiols do not function as surrogates of FDX2 and Fdx because they function differently (Parent et al., 2015; Gervason et al., 2019). Using a biochemical assay to detect and quantify protein-bound persulfide, the production of the sulfide ions in thiol-based reactions was shown to result from the cleavage of the persulfide carried by NFS1 (Figure 2B, red chart), whereas it is the persulfide of ISCU that is cleaved in FDX2-based reactions (Figure 2B, blue chart) (Parent et al., 2015; Gervason et al., 2019, 2021). Other thiols, including biological ones such as cysteine and GSH, were also shown to cleave persulfide and to sustain Fe-S cluster synthesis, thus raising the possibility that these thiol-based reactions could be physiologically relevant (Parent et al., 2015; Lin et al., 2020). However, the intrinsic properties of the thiol-based reaction compared to the FDX2-based one indicated that this is not the case. The thiol-based reaction is much slower and is far less efficient than the FDX2-based one, with only about 5% of the sulfide ions actually incorporated as Fe-S clusters compared to 95% in the case of the FDX2-based reaction. This is due to the lack of confinement and coordination of sulfide production with the presence of iron in ISCU. In the thiol-based process, the sulfide ions are released in solution in a two-step process involving formation of a persulfidated thiol (Figure 2B, reaction 7) that is subsequently cleaved into sulfide by reacting with a second thiol molecule (Figure 2B, reaction 9) (Parent et al., 2015). Then, the sulfide ions react with the iron center in ISCU to generate a Fe-S cluster *via* a still undefined mechanism (Figure 2B, reaction 10). These successive reactions are much slower and less efficient than the FDX2-based process which relies on concerted processes. Moreover, under physiological conditions, it is likely that the sulfide ions will diffuse outside of the NFS1-ISCU complex and thus the chance to specifically form a Fe-S cluster in ISCU will be very low. In contrast, in the FDX2-based reaction, persulfide supply to ISCU and sulfide production are coupled to the presence of iron in ISCU (Figure 2B, blue chart). Under these conditions, no “free” sulfide is produced. Moreover, in contrast to the FDX2-based reaction, the thiol-based one leads to the formation of [4Fe4S] clusters in addition to [2Fe2S] clusters (Colin et al., 2013; Parent et al., 2015), with DTT enhancing formation of [4Fe4S] clusters (Fox et al., 2015), a function that is attributed to the ISCA scaffold proteins (Muhlenhoff et al., 2011; Beilschmidt et al., 2017; Gervason et al., 2019; Weiler et al., 2020), which further questioned its physiological relevance. In conclusion, although biological thiols could play the role of DTT *in vivo*, the thiol-based reaction is not physiologically relevant for Fe-S cluster biosynthesis, which rules out the idea that FXN stimulates Fe-S cluster biosynthesis by enhancing the cleavage of NFS1’s persulfide by thiols.

## Frataxin as a Kinetic Activator of Persulfide Transfer to ISCU

### Biochemical Assays Point to an Acceleration of Persulfide Transfer by Frataxin

To establish the physiological function of FXN in Fe-S cluster biogenesis, the FDX2-based reaction was studied in a stepwise manner (Gervason et al., 2019). First, the *in vitro* reconstitution of the complete mouse ISC machinery including FDX2 (FDX2-based reaction) showed that FXN accelerates the formation of [2Fe2S] clusters. By analysing the effect of FXN at each step, we found that FXN accelerates the transfer of persulfide from NFS1 to ISCU (Figure 2B, reaction 4) (Parent et al., 2015; Gervason et al., 2019). An independent study also reported that human FXN facilitates sulfur transfer to ISCU, which points to a conserved mechanism across species (Bridwell-Rabb et al., 2014). However, Fe-S cluster assembly is a multi-step process, therefore to stimulate the whole process FXN must operate on the rate-limiting step. Persulfide transfer to ISCU was identified as the rate-limiting step in the mouse system, thereby, the stimulatory effect of FXN was directly correlated to its global stimulatory effect on the Fe-S cluster assembly process (Gervason et al., 2019). In other studies, FXN and its yeast homolog were found to stimulate the formation of the persulfide by NFS1 (Figure 2B, reaction 8), which suggest that FXN could operate at a different step of the reaction (Pandey et al., 2013; Patra and Barondeau, 2019). However, persulfide formation is not rate-limiting since kinetic studies showed that it is much faster than persulfide transfer (Parent et al., 2015; Gervason et al., 2019), which dismisses the possibility that FXN could stimulate Fe-S cluster assembly by accelerating persulfide formation on NFS1. Altogether, these mechanistic data have provided a long-awaited clarification on the functional role of frataxin by establishing that FXN stimulates Fe-S cluster biosynthesis by accelerating persulfide transfer.

Another important feature of the stimulation provided by FXN is that it accelerates the reaction and does not modify the nature of the Fe-S cluster formed, i.e., a [2Fe2S] cluster, nor the final yield of Fe-S clusters (Gervason et al., 2019), which fits the definition of an enzyme. However, since the reaction is already efficient in its absence, its functional role is best defined as an accelerator. This function is also consistent with the cellular phenotypes of yeast and mammalian cells lacking FXN, in which Fe-S clusters are still produced but at a lower level (Puccio et al., 2001; Duby et al., 2002; Muhlenhoff et al., 2002). Moreover, a number of transcription factors regulates FXN expression (Oktay et al., 2007; Guccini et al., 2011; Yandim et al., 2013; Fernández-Frías et al., 2020), which suggests that FXN could operate as a regulator to fine-tune Fe-S cluster biogenesis in response to variations in physiological conditions. Among them are Nrf2, the main regulator of the antioxidant defense (Sahdeo et al., 2014). Activation of Fe-S cluster production in response to reactive oxygen species (ROS) *via* the Nrf2-FXN axis could help restore Fe-S proteins damaged by ROS (D’Autreaux and Toledano, 2007). FXN is also part of the hypoxic response with both HIF-1α and HIF-2α activating FXN expression under hypoxic conditions (Oktay et al., 2007; Guccini et al., 2011). The activation of FXN expression under hypoxia might help improve mitochondrial bioenergetics under low oxygen concentration. The HIF-FXN axis also protects cells against ischemia-reperfusion episodes and is possibly critical for tumor progression (Amaral and Okonko, 2015; Nanayakkara et al., 2015; Schultz et al., 2016). Interestingly, the expression of FXN was found to be upregulated under iron-rich conditions and conversely downregulated under iron-starved conditions (Li et al., 2008). Thus, Fe-S cluster production seems to increase concomitantly with the level of available iron *via* modulation of FXN expression, which will amplify iron utilization by this pathway and thus prevent iron accumulation. In erythroid cells, the expression of FXN is not significantly modulated by the iron level (Becker et al., 2002), possibly to favor redirection of iron for heme biosynthesis. Therefore, the iron-dependent regulation of FXN expression might be an important regulatory circuit for iron utilization in different cellular contexts. Further studies in this direction will help better define the physiological role of FXN.

### Mechanism of Persulfide Transfer Stimulation by Frataxin

To move toward the design of drugs that could replace FXN, the next step is the elucidation of the mechanism by which FXN stimulates persulfide transfer. A key feature of this reaction is its iron-dependency, which is unique to this system (Gervason et al., 2019). Persulfide transfer reactions have been described in other sulfur insertion processes, such as the thiolation of transfer RNA, the synthesis of the molybdenum cofactor of nitrogenase and thiamine synthesis, but none of them are known to be metal-dependent (Black and Dos Santos, 2015; Leimkühler et al., 2017). In contrast, in the absence of iron in ISCU, the transfer of persulfide between NFS1 and ISCU and its acceleration by FXN are abolished (Gervason et al., 2019). This iron dependency thus appears as a central feature of Fe-S cluster biosynthesis, probably to couple sulfur acquisition by ISCU with iron availability. Thereby, understanding the role of the iron center in persulfide transfer is critical to unravel the mechanism by which FXN stimulates this reaction. Iron binds in the form of a ferrous iron (Fe^2+^) to the assembly site of ISCU and site directed mutagenesis suggests that the cysteine Cys35 and Cys61, the aspartate Asp37 and the histidine His103 are ligating the metal ion while Cys104, the persulfide receptor, remains unbound (Gervason et al., 2019). Unfortunately, no structure of iron-bound ISCU alone or within the NFS1-ISD11-ACP-ISCU-FXN complex has yet been reported to start assessing the structural details of persulfide transfer. A number of structures were solved but with zinc instead of iron, hereafter referred to as Zn-ISCU (Boniecki et al., 2017; Fox et al., 2019). However, since Zn-ISCU also enables persulfide transfer and its acceleration by FXN (Gervason et al., 2019), the structures solved with zinc should also be informative on the mechanism.

**FIGURE 4:**
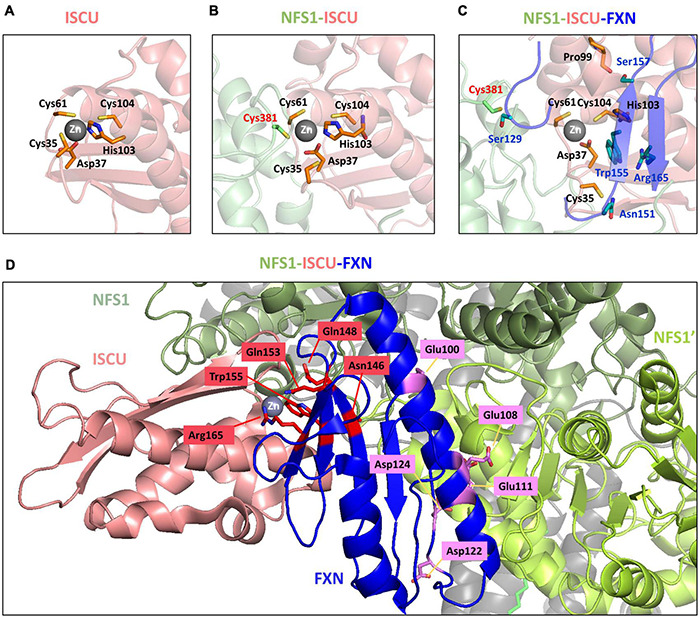
Structural rearrangement at the zinc site of ISCU upon binding of NFS1 and frataxin (FXN). Zinc site of panel **(A)** mouse ISCU (PDB code 1WFZ), **(B)** human NFS1-ISD11-ACP-ISCU complex (PDB code 5WLW) and **(C)** human NFS1-ISD11-ACP-ISCU-FXN complex (PDB code 6NZU). **(D)** FXN variants with impaired activity mapped on the structure of the human NFS1-ISD11-ACP-ISCU-FXN complex (PDB code 6NZU). The amino acids in red are involved in the interaction with ISCU, amino acids in purple are involved in interaction with NFS1. ISCU is colored in salmon, the two subunits of the NFS1 dimer are in light green (NFS1) and dark green (NFS1’), FXN is in blue.

The NMR structure of mouse Zn-ISCU (PDB code 1WFZ) revealed a structural arrangement very similar to the one suspected for Fe-ISCU (Figure 4A). In this structure, the zinc ion is coordinated by the cysteine Cys35 and Cys61, the aspartate Asp37 and the histidine His103, while Cys104, the persulfide receptor, remains unbound but at a close distance. The crystal structure of the human NFS1-ISD11-ACP-Zn-ISCU complex shows that the catalytic cysteine of NFS1 is binding to the zinc ion through exchange with Cys35 (Figure 4B; Boniecki et al., 2017). Although the structure was not solved with a persulfidated cysteine, it is likely that the persulfide carried by NFS1 would bind in a similar fashion. This might facilitate persulfide transfer by providing an electrophilic nature to the terminal sulfur of the persulfide (also called sulfane sulfur) to allow a nucleophilic attack by Cys104. However, this structure also highlights the steric hindrance between the catalytic cysteine of NFS1 and Cys104 due to the presence of the zinc ion and its ligands. It is likely that a structural rearrangement is needed to allow persulfide transfer. Interestingly, the structure of the pentameric NFS1-ISD11-ACP-Zn-ISCU-FXN complex solved by cryoelectron microscopy showed that FXN induces a rearrangement of the coordination sphere of the zinc ion (Figure 4C; Fox et al., 2019). FXN interacts with Cys35 of ISCU *via* its asparagine Asn151 and its glutamine Gln148, which stabilizes Cys35 in an unbound position away from the metal center. A second interaction involves tryptophan Trp155 of FXN interacting with His103 of ISCU *via* a π-π stacking interface that is anchoring His103 in an unbound position away from the zinc center. A third interaction between the serine Ser157 of FXN and the main chain proline Pro99 of ISCU is twisting the α-helix carrying Cys104 and His103, which may further stabilize the (FXN) Trp155-His103(ISCU) interaction and concomitantly promote the motion of Cys104 toward the metal ion. The structural rearrangement induced by FXN thus seems to clear access between the catalytic cysteine of NFS1 and Cys104 of ISCU and to force Cys104 into closer proximity to the zinc center. Altogether, this might facilitate direct persulfide transfer to Cys104. Importantly, Trp155 is strictly conserved among frataxin proteins (Supplementary Figure 1), which strengthens the idea that the His103-Trp155 interaction is a central feature of the activation mechanism by FXN.

Frataxin also interacts with the flexible loop of NFS1 and stabilizes the catalytic Cys381 halfway between the PLP of NFS1 and ISCU, which is difficult to rationalize with the stimulation of persulfide transfer (Fox et al., 2019). However, one has to consider that the catalytic cysteine is not persulfidated in this structure and thus this position may correspond to an intermediate state of the flexible loop returning to the PLP pocket for persulfide loading.

### Structure-Function Correlation With Clinical Variants

The analysis of point mutations in FXN provide further insight on the hypothetical mechanism proposed above. A small percentage of FRDA patients are heterozygous and contain, besides the GAA-triplet expansion, a point mutation in the *Fxn* gene (Santos et al., 2010; Galea et al., 2016). Two mouse models harboring the point mutations G127V and I151F, equivalent to G130V and I154F in human FXN, respectively, and recapitulating FRDA phenotypes were recently generated (Fil et al., 2020; Medina-Carbonero et al., 2022), which also helps better understand the impact of these mutations. These clinical variants affect residues that are conserved and important for structure and/or function of FXN (Table 1, clinical variants). In addition to clinical variants, other mutations impairing FXN and Yfh1 activities *in vivo* have been identified (Table 1, other mutants; Leidgens et al., 2010; Schmucker et al., 2011). Hereby, it is important to distinguish buried residues from those exposed to the solvent on the surface. Buried residues participate in the stability of the whole protein fold or may alter the maturation process (Table 1, core amino acids), whereas surface exposed residues might be involved in interactions with NFS1 and ISCU (Table 1, last two columns). Interestingly, the variants of surface exposed residues belong to two different domains of FXN within the NFS1-ISCU-FXN complex: the region spanning Glu96 to Asp124, corresponding to the first α-helix of FXN that is involved in the interaction with NFS1, and the region spanning Asn146 to Arg165 composing the β-sheet that is involved in the interaction with ISCU (Figure 4D). All the amino acids involved in the interaction with ISCU (N146, Q148, Q153, W155, and R165) are oriented toward the zinc center and are likely involved in the stimulation of persulfide transfer. The tryptophan Trp155 was already identified in the interaction with His103 of ISCU (Figure 4C). The asparagine Asn146, glutamine Gln153 and arginine Arg165 are packing close to the Trp155-His103 interaction and further stabilize it (Figure 4D). The glutamine Gln148 seems to cooperate with Asn151 to stabilize Cys35 of ISCU in the unbound position (Figure 4D). The analysis of the variants thus strengthen the idea that maintaining Cys35 and His103 in free positions, away from the metal center, are two critical structural features of the interaction between FXN and ISCU that promotes persulfide transfer.

**TABLE 1:** Key amino acids of FXN identified as impaired variants.

Position	Clinical variant	Other mutants	Core	Interaction with NFS1	Interaction with ISCU	References
Y95		Y95G	+			Schmucker et al., 2011
E100	E100R			+		Santos et al., 2010
L106	L106S		+			Santos et al., 2010
E108		E108K		+		Foury et al., 2007; Schmucker et al., 2011
E111		E111K		+		Foury et al., 2007; Schmucker et al., 2011
Y118	Y118X		+			Santos et al., 2010
D122	D122Y			+		Santos et al., 2010; Schmucker et al., 2011
Y123	Y123D		+			Galea et al., 2016
D124		D124K		+		Foury et al., 2007; Schmucker et al., 2011
G130	G130V		+			Calmels et al., 2009; Santos et al., 2010
N146	N146K	N146A			+	Santos et al., 2010; Schmucker et al., 2011
Q148	Q148R				+	Santos et al., 2010
Q153	Q153H	Q153A			+	Leidgens et al., 2010; Boll et al., 2021
I154	I154F		+			Calmels et al., 2009; Santos et al., 2010; Schmucker et al., 2011
W155	W155R	W155A			+	Santos et al., 2010; Schmucker et al., 2011
L156	L156P		+			Santos et al., 2010; Galea et al., 2016
S161	S161A		+			Leidgens et al., 2010
R165	R165C/P/D/N	R165A			+	Leidgens et al., 2010; Santos et al., 2010
W173	W173G		+			Santos et al., 2010; Schmucker et al., 2011
L182	L182F/H		+			Santos et al., 2010
H183	H183R		+			Santos et al., 2010
L186	L186R		+			Santos et al., 2010
L190	L190P		+			Galea et al., 2016
L198	L198R		+			Santos et al., 2010

*The table lists the amino acid positions identified as variants of FXN with impaired activity, both clinical variants of human FXN and those specifically designed based on the structures in mouse FXN and yeast Yhf1 (Leidgens et al., 2010; Santos et al., 2010; Schmucker et al., 2011). The roles of these amino acids are gathered into three different categories based on structural analysis: the core amino acids that are essential for the folding/stability of the protein and those involved in the interactions with NFS1 and ISCU.*

## Conclusion

Despite great efforts in elucidating the underlying mechanisms leading to the cellular phenotypes in FRDA patients and the search for therapeutic treatments, no cure is currently available. One of the main obstacles has remained the lack of understanding of the biochemical function of frataxin, but recent advances are opening new perspectives. The first models proposed that frataxin operates as an iron storage or iron chaperone protein, but these hypotheses were weakened by several studies disproving the ability of frataxin to store and deliver iron. Instead, frataxin was recently shown to stimulate Fe-S cluster biosynthesis by accelerating persulfide transfer from NFS1 to the scaffolding protein ISCU (Bridwell-Rabb et al., 2014; Parent et al., 2015; Gervason et al., 2019). Early exploration of phenotypes in patients as well as in cellular and animal models provided a starting point to target the symptoms appearing in the course of the disease, mainly mitochondrial dysfunction, iron accumulation, oxidative stress and lipid oxidation. Most of the drug candidates targeting these metabolic defects did not lead to significant improvement in clinical trials and were withdrawn, but some of them are still under studies (a list of current and withdrawn candidates is available at the FARA website^1^) (Lynch and Farmer, 2021). However, by acting exclusively on one of the multiple metabolic defects, these strategies are expected to provide only limited benefit, and compensatory effects might mitigate their therapeutic potential. These approaches are likely to be more potent in combination with each other. Among these strategies, iron chelation was shown to alleviate some of the cardiac phenotypes but was withdrawn due to worsening of the disease. Based on the assumption that frataxin may behave as a Ferritin-like iron storage or general iron donor, iron chelators such as deferiprone were developed to remove iron deposits in cardiomyocytes. Although deferiprone improved cardiac functions in clinical trials, adverse effects and worsening of the disease appeared at high doses (>20 mg/kg/day) (Pandolfo et al., 2014; Lynch and Farmer, 2021). The underlying reasons for the deleterious effects of direct iron chelation are complex. The recent findings on the functional role of frataxin as a sulfur transfer accelerator shed some new light to seize the links between iron accumulation and frataxin deficiency. Under conditions of frataxin deficiency, as sulfur donation is slowed down, iron builds up because it can no longer be used. Iron does not accumulate due to overabundance as one might predict based on the iron storage functional model. Therefore, by sequestering available iron, iron chelators further reduce Fe-S cluster biogenesis in FRDA patients, which could worsen the disease. In conclusion, although iron chelation therapy has a beneficial impact on the cardiac phenotype by clearing iron deposits, it becomes toxic probably by worsening the Fe-S clusters synthesis shortage.

The most recent strategies are now targeting the lack of FXN, which is the primary defect of FRDA, as this is expected to have the highest therapeutic benefit (Yang et al., 2021). Three different approaches are currently under development: increase of frataxin expression, delivery of recombinant frataxin protein and restoration of the frataxin locus by gene therapy. These strategies are highly promising. However, concerns regarding gene dosage will have to be considered with gene therapy and modulation of frataxin expression since frataxin overexpression was reported to be toxic in several models (Navarro et al., 2011; Wang et al., 2014; Vannocci et al., 2018; Belbellaa et al., 2020).

### Future Perspectives

In addition to the strategies restoring frataxin levels, the current knowledge on the functional role of frataxin in the stimulation of sulfur donation to ISCU opens new perspectives for the development of alternative approaches. For instance, sulfur donors and drugs substituting frataxin in sulfur transfer acceleration might be of interest in the treatment of FRDA. In this context, unraveling the mechanism by which frataxin overexpression is toxic will become a key question to solve in the near future in order to adjust therapies based on frataxin substitution and the modulation of its expression.

## Author Contributions

BD’A, BM, and KW wrote the review. SG performed the protein alignments and provided the Figure 1. All authors contributed to the article and approved the submitted version.

## Conflict of Interest

The authors declare that the research was conducted in the absence of any commercial or financial relationships that could be construed as a potential conflict of interest.

## Publisher’s Note

All claims expressed in this article are solely those of the authors and do not necessarily represent those of their affiliated organizations, or those of the publisher, the editors and the reviewers. Any product that may be evaluated in this article, or claim that may be made by its manufacturer, is not guaranteed or endorsed by the publisher.

## Footnotes

1
https://www.curefa.org/pipeline
