# Supplementary material for: Recent Advances in the Elucidation of Frataxin Biochemical Function Open Novel Perspectives for the Treatment of Friedreich’s Ataxia
Source: Front Neurosci. 2022 Mar 2;16:838335. doi: 10.3389/fnins.2022.838335 (PMC8924461; doi:10.3389/fnins.2022.838335)
Supplement: Supplementary file 1 [file Image_1.pdf]

Supplementary Material

Supplementary Figures

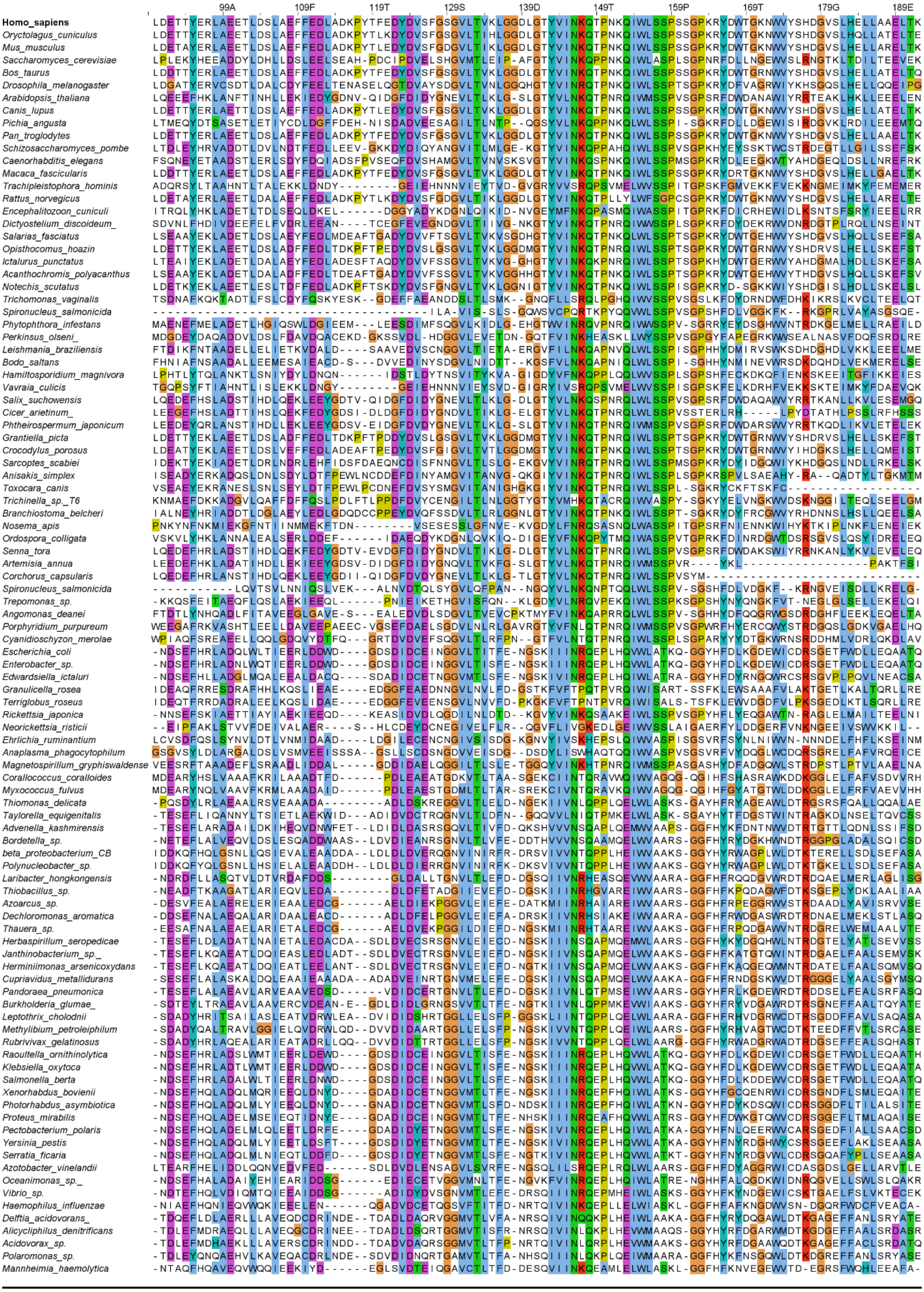

Supplementary Figure 1: Multiple alignments of eukaryotic and prokaryotic frataxins

FXN homologs (50 eukaryotes and 50 prokaryotes) were aligned using Jalview v2.11.1.4. The alignment start to the residue 90 and end to the residue 192 based on the numbering of human FXN. The colour are based on Clustal X nomenclature where conserved hydrophobic residues (A, I, L, M, F, W, V) are in blue, conserved positively charged residues (K, R) in red, conserved negatively charge residues (E, D) in magenta, conserved polar residues (N, Q, S, T) in green, glycine in orange, proline in yellow, conserved aromatic residues (H,Y) in cyan and non-conserved residues in white.
